# Anatomy Education and Training Methods in Oral Surgery and Dental Implantology: A Narrative Review

**DOI:** 10.3390/dj12120406

**Published:** 2024-12-12

**Authors:** Carlo Barausse, Pietro Felice, Roberto Pistilli, Gerardo Pellegrino, Lorenzo Bonifazi, Subhi Tayeb, Irene Neri, Foteini-Dionysia Koufi, Antonietta Fazio, Maria Vittoria Marvi, Lucia Manzoli, Stefano Ratti

**Affiliations:** 1 Cellular Signalling Laboratory, Anatomy Center, Department of Biomedical and Neuromotor Sciences (DIBINEM), University of Bologna, 40126 Bologna, Italy; irene.neri3@unibo.it (I.N.); foteinidionysi.koufi@unibo.it (F.-D.K.); antonietta.fazio2@unibo.it (A.F.); mariavittoria.marvi2@unibo.it (M.V.M.); lucia.manzoli@unibo.it (L.M.); stefano.ratti@unibo.it (S.R.); 2 Unit of Oral Surgery, Department of Biomedical and Neuromotor Sciences (DIBINEM), University of Bologna, 40125 Bologna, Italy; pietro.felice@unibo.it (P.F.); gerardo.pellegrino2@unibo.it (G.P.); lorenzo.bonifazi2@unibo.it (L.B.); subhi.tayeb@studio.unibo.it (S.T.); 3 Unit of Oral and Maxillofacial Surgery, San Camillo-Forlanini Hospital, 00152 Rome, Italy; r_pistilli@libero.it

**Keywords:** human anatomy, education, training, oral surgery, dental implants

## Abstract

**Background:** Oral and implant surgery represent highly specialized fields within dentistry that require a deep understanding of complex anatomical structures, together with practical hands-on experience. The present review examines common trends in oral and implant surgery training, focusing on how traditional methods like donated body dissection coexist with different and modern educational tools, and highlights the pros and cons of the different approaches in order to optimize training outcomes. **Methods:** A systematic literature search was carried out using the databases PubMed and Cochrane Library including the last 10 years of published articles about training in oral surgery and implantology. Starting from a total of 1319 studies, 47 were included to be carefully evaluated, and 20 studies were finally selected for this narrative review. The studies utilize methodologies such as randomized controlled trials (RCTs), cross-sectional surveys, case–control studies, and systematic reviews. The results were thematically organized, highlighting key quantitative outcomes and drawing connections between the different educational approaches. **Results:** From the narrative review, it emerged that oral and implant surgery training requires a careful balance between traditional methods, such as donated human body dissection, and modern technological advancements like virtual simulations and synthetic models. While animal and synthetic models have specific uses, their application remains limited in replicating the full complexity of human anatomy. These last technologies offer flexibility and expanded access to education but do not substitute for the hands-on experience gained through donated human body dissection. **Conclusions:** As educational institutions continue to evolve their training programs, ensuring access to human body dissection remains of paramount importance. Combining the strengths of both traditional and modern approaches may help optimize oral and implant surgery education, enhancing student preparedness without overlooking the critical value of direct anatomical experience.

## 1. Introduction

Oral and implant surgery represent highly specialized fields within dentistry that require a deep understanding of complex anatomical structures, such as nerves, muscles, and blood vessels in the oral and maxillofacial region. These specialties continue to evolve as new surgical techniques and technologies emerge, requiring from the oral surgeon not only theoretical knowledge and updates, but also practical skills and training. Education in oral surgery and implantology thus needs to strike a balance between solid comprehension, practical hands-on experience, and exposure to cutting-edge surgical advancements. Globally, there are different approaches to training in these fields, but the fundamentals of anatomy education remain critical for all surgeons.

Historically, anatomy education has relied on traditional methods like textbooks and, most importantly, donated body dissection, which provides trainees with an immersive, three-dimensional understanding of human anatomy. This method allows future oral surgeons and dental implantologists to physically explore anatomical structures, giving them the spatial awareness and tactile experience necessary for mastering complex surgical techniques. Moreover, human body dissection is extremely valuable for first-time attempts and the development of novel surgical approaches [1]. To make education more accessible, alternative complementary approaches such as animal and synthetic models can be integrated into oral and dental implant surgery training.

Moreover, in recent years, advances in virtual simulation, tele-didactics, and other digital learning platforms have supplemented traditional training methods [2]. However, the tactile and immersive learning experience provided by donated body dissections plays a crucial role in anatomical education, particularly for surgical procedures.

This review examines common trends in oral and implant surgery training, focusing on how traditional methods like donated body dissection coexist with different and modern educational tools such as virtual reality (VR), synthetic models, and animal models, and highlights the pros and cons of the different approaches in order to optimize training outcomes.

## 2. Materials and Methods

This narrative review was conducted to evaluate the educational tools and methodologies employed in oral surgery and implantology training. The review followed the applicable elements of the Preferred Reporting Items for Systematic Reviews and Meta-Analyses (PRISMA) guidelines to ensure transparency and methodological rigor.

### 2.1. Search Strategy

A systematic literature search was carried out using the databases PubMed and Cochrane Library. The search was conducted on 4 September 2024, covering the period from 2014 to 2024. The search strategy combined the following terms: (“*oral surgery*” OR “*implantology*”) AND (“*education*” OR “*training*”) AND (“*undergraduate*” OR “*postgraduate*” OR “*master*” OR “*clinical training*” OR “*dental education*”). These terms were chosen to capture studies exploring diverse educational methods in oral and implant surgery training for both undergraduate and postgraduate levels.

### 2.2. Inclusion and Exclusion Criteria

The inclusion criteria for the review were as follows: studies published between 2014 and 2024, written in English, and focusing on oral surgery and/or implantology education. Only studies discussing practical and clinical training tools, such as donated human body dissection, synthetic models, and virtual simulations, were included. Studies were excluded if they were published before 2014, lacked access to the full text, or focused exclusively on theoretical education without practical components. Any duplicate articles across databases were also removed.

### 2.3. Article Selection Process

**Figure 1:**
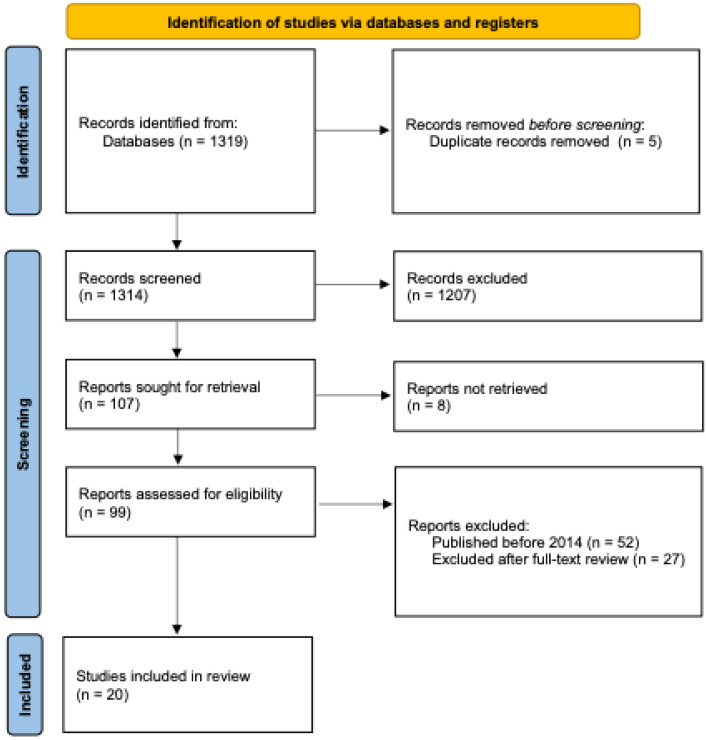
This diagram illustrates the systematic process of identifying, screening, and selecting studies for inclusion in this narrative review.

The search yielded a total of 1319 studies. After removing 5 duplicates using EndNote 21 Software (Clarivate, Philadelphia, PA, USA), 1314 studies remained. The titles and abstracts were screened according to the inclusion and exclusion criteria, and 1207 articles were excluded due to their lack of relevance to practical or clinical training in oral surgery. This left 107 articles for further evaluation. Of these, 8 articles were eliminated because the full text was not available for review. According to PRISMA guidelines, we attempted to contact the authors to obtain the missing full texts and allowed a response period of two weeks. However, no responses were received, and these articles were therefore excluded from the review. The remaining 99 articles were carefully assessed, and 52 were excluded because they were published before 2014. Of the 47 articles that met the initial criteria, an additional 27 were excluded after full-text review because they did not align with the inclusion criteria for practical or clinical relevance. Ultimately, 20 studies were selected for inclusion in this narrative review. (Figure 1) The screening of the articles was made by two oral surgeons and implantologists with expertise in research, and when there was no agreement between them, the opinion of a third dentist with the same background was asked for.

### 2.4. Data Extraction and Quality Assessment

Data extraction was conducted systematically from the selected studies, focusing on the educational tools evaluated, the context of their application, and the reported outcomes such as procedural accuracy, student engagement, and knowledge retention. Although a formal risk of bias assessment was not undertaken, studies were critically reviewed based on design, methodology, and outcome relevance. While this was a narrative review, methodological rigor was applied to interpret the results thoughtfully.

## 3. Results

This systematic review includes 20 studies published between 2014 and 2024, which examine various educational strategies and tools in oral surgery and implantology training. The studies utilize methodologies such as randomized controlled trials (RCTs), cross-sectional surveys, case–control studies, and systematic reviews. The results are organized thematically below, highlighting key quantitative outcomes and drawing connections between the different educational approaches.

### 3.1. Simulation-Based Learning

Simulation-based learning emerged as a central theme in many studies, with significant improvements reported in both student confidence and technical proficiency. Buchbender et al. (2021) [3] evaluated an oral surgery simulator (Kobra Surgery Simulator, Forsslund Systems, Sundbyberg, Sweden), showing that although students displayed improved surgical precision with practice, their performance remained statistically similar to that of experienced clinicians. Third-year students, in particular, removed more soft tissue than fourth-year students, indicating lower precision. While both students and clinicians found the simulator useful for skill development, participants generally favored conventional plastic models for their superior tactile feedback. Despite this, the Kobra simulator was viewed as a valuable complementary tool for surgical training. However, it should be noted that the study involved only a single training session, which may influence the extent of skill development observed. Additionally, the study’s small sample size may limit the generalizability of its findings, suggesting that further research with larger cohorts could provide more robust conclusions about the simulator’s effectiveness. Similarly, Yoshida et al. (2022) [4] investigated the use of 3D simulation software paired with printed models in osteotomy training. The study found a significant improvement in student performance, with test scores in the self-simulation group increasing from 15.5 to 17.8 points post training. Additionally, students in this group showed a deeper understanding of surgical techniques and the use of instruments, with statistically significant improvements in both areas (*p* < 0.01). Participants also reported that the self-simulation helped them better comprehend 3D anatomical relationships, boosting their confidence in performing surgical procedures. However, the study’s reliance on self-reported questionnaires and short-term test scores may not fully capture the development of practical skills or deeper learning, suggesting that future research could benefit from incorporating more objective and long-term assessments. Shetty et al. (2023) [5] also showed the effectiveness of fully guided virtual implant planning software (VIPS), (Romexis, version 6.2.1, Planmeca Viso 7 CBCT unit, Finland) in improving both procedural accuracy and student engagement. Students who received hands-on training with VIPS, alongside didactic lectures and video instruction, showed significantly higher procedural competence, scoring 4.30 ± 0.70 out of 5, compared to 3.17 ± 1.02 for the lecture-based group and 2.97 ± 1.25 for the video-based group (*p* < 0.01). Additionally, 94.44% of students strongly agreed that VIPS made implant planning easier to understand, and 88.88% reported that they liked the VIPS training sessions. These results highlight the substantial improvement in both accuracy and learning experience when hands-on virtual tools are integrated into implant planning education. Extending this, Coffey-Zern et al. (2015) [6] explored the use of simulation in oral and maxillofacial surgery residency programs, reporting a marked increase in resident confidence, particularly in managing emergency scenarios. The program included multiple simulation sessions covering various critical areas such as difficult airway management, ACLS (Advanced Cardiovascular Life Support) training, laparoscopic fundamentals, and delivering difficult news. One notable simulation exercise focused on proper local flap elevation, rotation, and suturing using a pig cadaver model, providing hands-on experience in soft tissue management. Each session was structured with 3 h of practical simulation followed by a 1 h debriefing and didactic discussion. This simulation-based approach proved especially effective in enhancing the preparedness of residents for high-risk clinical situations, further underscoring the relevance of simulations in surgical education. Moreover, Zhang et al. (2020) [7] compared virtual and jaw simulations, concluding that combining these methods (V-J group) produced significantly higher implant precision and theoretical scores than using either method alone (*p* < 0.01). The simulations focused on preclinical implant training, including implant placement on pig mandibles and evaluating implant accuracy through cone-beam computed tomography (CBCT). This finding highlights the potential of integrating multiple simulation tools to maximize student performance in both theoretical knowledge and practical skills. However, the limited duration of the training sessions, totaling only 8 h over 4 days, may not be sufficient to evaluate the long-term impact on skill retention and clinical application.

### 3.2. 3D-Printed and Patient-Specific Models vs. Donated Human Body Models

In addition to virtual simulations, several studies examined the use of 3D-printed and patient-specific models, often comparing them with traditional human body models. Seifert et al. (2020) [8] conducted a study involving 38 fourth-year dental students comparing 3D-printed patient-specific models with traditional human bodies models in an oral and maxillofacial surgery curriculum. The study found that donated human body models were rated significantly higher for soft tissue realism (median score of 9 vs. 5 for 3D-printed models, *p* < 0.001). Students, moreover, appreciated the ability to mount 3D-printed models in phantom heads for realistic intraoral simulations but noted the need for improved durability of the silicone soft tissue. Complementing this, Hu et al. (2023) [9] explored the use of patient-specific 3D-printed models in immediate implant placement. The study found significant improvements in students’ understanding of surgical procedures, with median scores increasing from 6.4 to 8.6 for surgical procedure knowledge and from 5.7 to 8.4 for minimally invasive tooth extraction (*p* < 0.001). However, participants (30 students) noted the absence of soft tissue realism. While 3D models were highly effective for understanding bone anatomy and prosthetically driven implantology, the lack of soft tissue simulation limited their full applicability in surgical training. Supporting these findings, Watanabe et al. (2019) [10] examined the use of donated human body models preserved using a saturated salt solution (SSS) in oral surgical education. The study showed that SSS-embalmed donated human bodies provided superior soft tissue realism compared to plastic models, with participants reporting significant improvements in self-assessed confidence levels for procedures such as maxillary tuberosity bone harvesting (*p* = 0.002). The realistic texture and pliability of the soft tissues were critical for developing practical skills, particularly in soft tissue handling. These findings suggest that SSS-embalmed donated human bodies offer a more accurate and effective training model, better-preparing students for clinical practice. However, the study’s reliance on self-reported confidence levels as a measure of training effectiveness may not fully reflect actual surgical competency, suggesting that incorporating objective performance evaluations could provide a more accurate assessment.

In this context, while 3D-printed models are valuable tools for enhancing anatomical understanding, donated human body models continue to be regarded as the gold standard for training that involves both soft and hard tissue manipulation. The combination of these approaches, integrating 3D models for preliminary training and donated human body models for advanced practice, may offer the most comprehensive educational framework.

### 3.3. Blended and Online Learning Approaches

With the onset of the COVID-19 pandemic, online and blended learning approaches gained prominence [11,12]. Jiang et al. (2021) [13] surveyed 104 dental students and 57 resident physicians, finding that 78.9% of respondents were satisfied with online lecture-based learning and case-based learning. However, only 46.6% felt that these formats adequately addressed practical skills, underscoring the limitations of virtual-only education in surgical fields. The need for physical, hands-on experience was consistently highlighted, as the students noted the challenges in translating theoretical online learning into clinical practice. Blond et al. (2024) [14] conducted a randomized controlled trial comparing blended learning with traditional instruction methods in 73 fourth-year dental students. The study found that overall satisfaction was higher in the blended learning group, with 97.6% of students expressing satisfaction compared to 93% in the traditional learning group (*p* = 0.002). Additionally, the blended learning group rated the course’s duration and pace more favorably (*p* = 0.006) and felt better prepared for practical work (*p* = 0.016). Although both groups showed significant knowledge improvement, students in the blended group felt more confident in performing complex procedures, such as surgical tooth extraction (*p* = 0.043) and managing failed extractions requiring bone removal (*p* = 0.044). These results suggest that blended learning enhances student confidence and satisfaction, making it a valuable instructional method in dental education, albeit taking into consideration all of its possible drawbacks and the urgent and definitive need for practical sessions. However, the study’s lack of long-term evaluation means it remains unclear how well the skills and knowledge gained through online learning are retained and applied in clinical practice, suggesting that future research could explore these aspects to provide a more comprehensive understanding. Elhadidi et al. (2024) [15] found that hybrid learning models, which combined online content with in-person clinical exposure, were more effective than fully online approaches during the COVID-19 lockdown. In a cohort of 40 dental students, dissatisfaction with practical teaching dropped from 55% in 2020 to 30% in 2021 when hybrid learning was employed. Moreover, 93% of students in 2021 expressed overall satisfaction with the education system, compared to 65% in 2020, highlighting the continued importance of hands-on training, even within hybrid models.

### 3.4. Hands-On Training and Practical Experience

Hands-on training remains a critical component of clinical education, as emphasized by numerous studies. Fischer et al. (2023) [16] evaluated a novel implantology training program aimed at integrating implant surgery procedures into the dental curriculum. The program, consisting of four modules that covered both theoretical and practical content, was assessed through two questionnaires completed by 94 students. The analysis showed that the program was well received, with Cronbach’s alpha values exceeding 0.7 for all categories except skills training. Median scores ranged from 4.75 to 6, indicating high satisfaction. Significant Pearson correlations (*p* < 0.05) were found between key categories, including perceived importance, lectures, and tutor performance. In terms of student feedback, 88% expressed a desire for more practical exercises in dental implantology, and 35% suggested that implant procedures on real patients under supervision should be incorporated. While the program was highly accepted, 12% of students felt it did not fully prepare them for performing implants independently, highlighting the need for more extensive hands-on training. These results emphasize the importance of integrating more clinical practice into dental curricula to ensure students feel confident in performing surgical procedures. Bai et al. (2017) [17] further examined the role of problem-based learning (PBL) in dental alveolar surgery education. In a study of 90 dental undergraduates, PBL graduates rated their clinical preparedness significantly higher than those in the traditional lecture-based group (7.15 vs. 6.66, *p* = 0.044). Additionally, students in the PBL group demonstrated superior collaboration and problem-solving abilities, which were essential for applying theoretical knowledge to clinical scenarios. This study highlights the importance of active learning strategies, such as PBL, in fostering clinical competence and preparing students for the complexities of dental surgery. Bauer et al. (2016) [18] also emphasized the importance of hands-on training, showing that after a one-day practical lesson in surgical skills, the average test scores increased significantly (*p* < 0.05). Moreover, the percentage of students expressing interest in a surgical career rose substantially, with the average score for interest in surgery improving from 3.41 to 2.75 (*p* = 0.028). These data underscore the motivational impact of early hands-on training in fostering a potential surgical career path among medical students.

### 3.5. Educational Tools and Skill Acquisition

Advanced educational tools, such as interactive videos and flipped classroom models, have been shown to improve student learning outcomes. Mitov et al. (2020) [19] showed that the use of screencast training videos in virtual 3D implant planning significantly reduced task completion times for dental students and dentists (*p* < 0.001), with students completing tasks in 15.3 min compared to 22.8 min in the control group. Additionally, the screencast training improved procedural accuracy, with students in the experimental group showing a 3D deviation at the implant base of 1.38 mm compared to 2.9 mm in the control group. However, the study’s relatively small sample size, especially when divided into experimental and control groups (51 participants, divided into three groups: dental students (21), dental technicians (16), and dentists (16)), may limit the generalizability of its findings, suggesting that future research with larger cohorts could provide more robust conclusions. Bock et al. (2020) [20], in a study utilizing a flipped classroom model for oral surgery education, showed a significant improvement in student knowledge retention. Furthermore, 76.2% of students regularly used the e-learning platform for preparation, underscoring the role of digital tools in promoting independent study. Student feedback highlighted the flipped classroom’s effectiveness, with high satisfaction ratings for the interactive design and a positive influence on their motivation for further learning. These results emphasize the value of integrating digital tools and innovative teaching models to enhance both knowledge acquisition and long-term retention in clinical education.

### 3.6. Clinical Application and Student Competence

Several studies have highlighted the ongoing gap between theoretical knowledge and clinical competence in dental education (Table 1). Koole and De Bruyn (2014) [21] conducted a systematic review of 37 publications, showing that while most dental schools included theoretical implant education, clinical training was minimal. Barriers such as insufficient funding, limited staff availability, and restricted patient access hindered the broader implementation of hands-on training. Despite these limitations, implant dentistry education positively influenced students’ future clinical practice, with many reporting a high appreciation for the knowledge gained. The study underscores the importance of integrating more clinical practice into dental curricula to better prepare graduates for real-world challenges in implantology. Enabulele and Omo (2020) [22] reported that in Nigerian dental schools, the majority of programs place a strong emphasis on theoretical instruction, with 80% of schools incorporating dental implantology into their undergraduate curriculum. However, only 25% of these programs provide students with hands-on experience, and none require students to perform implant procedures themselves. This lack of clinical exposure was identified as a significant barrier to developing practical competence, as students are primarily limited to didactic lectures and, in rare cases, clinical demonstrations. Furthermore, 90% of the schools cited insufficient resources and a lack of industry support as major obstacles to offering more comprehensive implant training. Similarly, Van Assche et al. (2018) [23] emphasized the need for enhanced certification processes and standardized clinical training, particularly in implantology, where only 250 h of training, 60% theoretical and 40% clinical, are currently required for European certification. Both studies underscore the importance of integrating more comprehensive, practice-based training to ensure that graduates are better prepared for real-world clinical challenges. Barwacz et al. (2016) [24] echoed these concerns in their comparison of Canadian and U.S. predoctoral implantology programs, emphasizing the need for standardized clinical exposure to ensure students are adequately prepared for independent practice.

## 4. Discussion

Human body dissection has been central to understanding human anatomy and remains crucial in surgical education worldwide. It is invaluable not only in early education but also in ongoing surgical training to enhance skills and simulate complex procedures. Oral surgery, focusing on a highly intricate anatomical area rich in vital structures, particularly benefits from dissection. While various training tools exist, several studies in this review highlight that the dissection of donated human bodies remains a critical reference point. Watanabe et al. (2019) [10] highlighted the unique value of donated human body dissection in providing tactile feedback, particularly in soft tissue handling, making it very difficult to replace with other training methods.

Several alternatives to human body dissection have been proposed, the most common being animal and synthetic models. While these alternatives may offer some educational benefits, they seem to fall short of replicating the complexities of the human anatomy essential for oral surgery. Animal models, in particular, involve the use of sacrificed animals to simulate anatomical evaluation and surgical procedures. One ethical and cost-effective application of animal models in oral surgery training involves the use of porcine mandibles. These are often employed to simulate surgeries involving both bone and soft tissue, such as implant placement, connective tissue grafting, and guided bone regeneration. Porcine mandibles provide a reasonably accurate representation for trainees and are also utilized for suturing simulations. Using butcher’s waste parts makes this an ethical choice with reduced costs; however, its application is limited to a few surgical procedures. While findings suggest that porcine mandibles can offer some practical experience, they lack the anatomical precision needed for high-level and more complex oral and implant surgery simulations. Therefore, these models are unlikely to match the effectiveness of donated human bodies in replicating oral surgery scenarios.

Moreover, the use of animal models in medical training raises several ethical and practical concerns. One of the primary issues is the welfare of the animals themselves; even when efforts are made to minimize suffering, invasive procedures or experiments often involve significant distress and discomfort. This raises questions about the morality of subjecting living beings to harm for educational purposes, especially when alternative methods might be available.

In recent years, to address the challenges posed by limited donated human body availability, artificial models and technological aids have been explored as substitutes. Many universities now use synthetic models for their students due to their ease of use and availability. These models are particularly useful at the undergraduate level, where less complex topics are covered. Each synthetic model usually provides a specific type of simulation as shown by Hu et al. (2023) [9]. Furthermore, considering the growing need for hands-on training sessions, as highlighted by Bauer et al. (2016) [18], synthetic models offer valuable practical experience, helping students develop a fundamental understanding of human anatomy.

Despite advancements in synthetic models and virtual simulations, as suggested by Hu et al. (2023) [9] and Seifert et al. (2020) [8], traditional donated human body dissection continues to provide unmatched educational value, particularly in postgraduate training. While virtual and synthetic models offer valuable visual and theoretical understanding, they often lack the tactile realism and haptic feedback essential for mastering surgical skills. Limitations such as the absence of tissue resistance, texture variations, and the inability to simulate the complexities of human anatomical variations make these models less effective for advanced surgical training. While synthetic models may be beneficial for undergraduate courses, where exercises are generally less complex, for postgraduate training, the reliance on donated human body dissection becomes crucial. In oral surgery and implantology, it is difficult to replicate the complexity of human anatomy or provide trainees with comparable tactile feedback using artificial models alone [25]. However, virtual simulations, while more expensive to develop and implement initially, have proven cost-effective in the long term due to their scalability, the ability to track progress, and the reduced dependence on physical resources. A common application of synthetic models is in the simulation of tooth extractions, which effectively replicate the positioning of luxators and forceps in the mouth. Moreover, Zhang et al. (2020) [20] found that combining virtual and physical simulations may lead to higher precision in implant procedures, indicating that a hybrid approach could enhance student outcomes. While the integration of virtual and physical models offers significant benefits, it is important to consider potential challenges or downsides of this combined approach. One concern is the danger of relying too much on synthetic or virtual models, which may lack the anatomical variability found in real human bodies. This over-reliance could lead to “over-standardizing” training, potentially leaving students unprepared for the diverse anatomical presentations encountered in actual clinical practice. Additionally, certain tactile skills, such as suturing and soft tissue handling, are especially challenging to learn without practice on donated human bodies, highlighting why dissection remains valuable.

The accurate replicability of surgical procedures is also well integrated into virtual models, which provide valuable three-dimensional visualization of anatomical structures and are easy to access. Pre-surgical planning, particularly in oral surgery, often relies on virtual software programs for three-dimensional radiographic evaluation, making these technologies beneficial not only for educational purposes but also for clinical practice. Virtual simulations, when combined with hands-on training, as observed by Shetty et al. (2023) [5], seem to offer improvements in both theoretical understanding and procedural accuracy. Nonetheless, there is a risk that integrating multiple training methods without careful balance may lead to gaps in skill acquisition. For example, students might excel in virtual environments but struggle when confronted with the unpredictability of live surgery.

An innovative example of integrating traditional human body dissection with modern technologies is the full-body revascularized and ventilated specimen (SimLife^®^ technology, Poitiers, France) [26]. This advanced technology allows the donated human bodies to simulate complex surgical procedures with high levels of specialization. It has already been used to simulate maxillofacial surgeries, where the presence of bleeding temporal and cervical vessels was crucial, offering a remarkably realistic experience. Such advancements appear promising for oral surgery, especially in high-risk scenarios such as those involving hemorrhages. However, the inability to emulate fine bleeding in smaller vessels might remain a potential limitation in oral surgery and implantology [27,28]. To address these challenges, further research is needed to evaluate the specific impact of hybrid training models on surgical competency and patient outcomes. Future studies could focus on assessing how combining virtual simulations with traditional dissection influences the development of tactile skills, clinical decision making, and adaptability in real-world surgical settings. Understanding these factors will be crucial in optimizing educational strategies to produce competent and confident oral surgeons.

## 5. Conclusions

Oral and implant surgery training requires a careful balance between traditional methods, such as donated human body dissection, and modern technological advancements like virtual simulations and synthetic models. While animal and synthetic models have specific uses, their application remains limited in replicating the full complexity of human anatomy. These technologies offer flexibility and expanded access to education but do not fully substitute for the hands-on experience gained through donated human body dissection.

As educational institutions continue to evolve their training programs, ensuring access to essential resources like donated human body dissection will remain important. Combining the strengths of both traditional and modern approaches may help optimize oral and implant surgery education, enhancing student preparedness without overlooking the critical value of direct anatomical experience. However, further research is needed to assess the long-term impact of these combined methods on clinical outcomes.

## Figures and Tables

**Table 1:** This table provides an overview of the studies selected for inclusion in this narrative review. It outlines key information for each study [3,4,5,6,7,8,9,10,13,14,15,16,17,18,19,20,21,22,23,25].

Author	Year	Title	Study Design	Participants	Intervention	Main Outcomes	Key Findings	Limitations
Koole and De Bruyn	2014	Contemporary undergraduate implant dentistry education: a systematic review	Systematic Review	37 included publications	Implant dentistry education at the undergraduate level	Improvement in theoretical knowledge, limited clinical practice	Mainly theoretical training, limited clinical exposure	Variability among programs, lack of standardization
Coffey-Zern et al.	2015	Incorporating Simulation Into Oral and Maxillofacial Surgery Residency Education and Training	Descriptive Study	Oral and maxillofacial surgery residents	Simulation of surgical procedures	Increased resident confidence and competence, improved emergency management	Debriefing and post-simulation reflection highly valued by residents	Lack of long-term efficacy data
Bai et al.	2017	Follow-up assessment of problem-based learning in dental alveolar surgery education: a pilot trial	Randomized controlled trial (RCT)	90 dental undergraduates	Problem-based learning (PBL) in dental alveolar surgery	Better preparedness for clinical practice, improved collaboration skills	PBL group rated higher in clinical problem solving and collaboration skills compared to traditional teaching	Limited to one class and one dental school
Barwacz et al.	2016	Comparison of Canadian and United States Predoctoral Dental Implant Education	Cross-sectional survey	10 Canadian dental schools	Survey of implant education practices	Homogeneity in implant curricula across Canadian schools, majority use guided surgery planning	Half of Canadian directors feel students are not adequately prepared for implant therapy at graduation	Small sample size, lack of statistical association analysis
Bauer et al.	2016	Can a one-day practical lesson in surgical skills encourage medical students to consider a surgical career?	Prospective study	54 medical students	One-day practical training in maxillofacial surgical skills	Increased interest in surgical careers, improved surgical knowledge	Significant increase in interest in maxillofacial surgery after training	Short follow-up period, limited to one-day intervention
Seifert et al.	2020	3D-printed patient individualised models vs. donated human bodies models in an undergraduate oral and maxillofacial surgery curriculum: Comparison of student’s perceptions	Controlled cohort study	38 fourth-year dental students	Comparison between 3D-printed patient models and donated human body models for oral surgery training	3D models better for anatomical correctness, donated human bodies models better for soft tissue feedback	3D models were cost-efficient and provided better anatomical simulation, but less realistic soft tissue	Limited sample size, short-term study
Van Assche et al.	2018	Guidelines for development of Implant Dentistry in the next 10 years	Consensus paper	40 junior scientists and clinicians	Discussion on future trends in implant dentistry	Guidelines on certification, continuing education, innovation, and societies	Emphasis on the need for improved certification, dental associations’ role, and technological innovations	Based on consensus without detailed empirical data
Watanabe et al.	2019	The Usefulness of Saturated Salt Solution Embalming Method for Oral Surgical Education	Prospective study	22 participants including oral surgeons, residents, and dentists	Saturated salt solution embalming (3S) method for oral surgical education	3S models were effective in preserving anatomical structures for surgical practice	Students reported high satisfaction with the anatomical realism of 3S models	Limited to a single institution and sample size
Zhang et al.	2020	Virtual versus jaw simulation in Oral implant education: a randomized controlled trial	Randomized controlled trial (RCT)	80 s- and third-year dental students	Comparison between virtual simulation and jaw simulation for oral implant education	V-J and J-V groups showed better implant precision and higher theoretical scores	The combination of virtual and jaw simulations improved student performance in both theory and practical skills	Short training duration, limited to one institution
Mitov et al.	2020	Use of interactive instructional tools in virtual 3D planning	Experimental study	51 dental students, dental technicians, and dentists	Screencast training videos for virtual 3D implant planning	Screencast videos improved planning speed and quality, especially for students and dentists	Screencast training was more beneficial for less experienced users	Small sample size and limited to one software tool
Enabulele and Omo	2020	Teaching of dental implantology to undergraduate dental students: The Nigerian experience	Cross-sectional descriptive study	10 Nigerian dental schools	Survey on implantology teaching practices	Mostly theoretical teaching with limited clinical exposure	Lack of clinical competence; insufficient resources and industry support; plans for improvement	Limited to Nigeria, no direct clinical training for students
Jiang et al.	2021	Online dental teaching practices during the COVID-19 pandemic: a cross-sectional online survey from China	Cross-sectional survey	104 dental students, 57 resident physicians	Online dental education during COVID-19 using LBL, CBL, PBL, TBL, and RBL	LBL and CBL were preferred teaching methods; high student satisfaction with online teaching	Lecture-based learning (LBL) and case-based learning (CBL) were the preferred teaching methods among students compared to problem-based learning (PBL), research-based learning (RBL), and team-based learning (TBL); overall, 78.9% of the students expressed satisfaction with the online classes	Limited to one institution, self-reported data, no long-term follow-up on effectiveness
Yoshida et al.	2022	Osteotomy training for dental students using three-dimensional simulation software and maxillofacial 3D-printed models	Cross-sectional study	24 5th-year dental students	Maxillofacial simulation software (MSS) + 3D-printed models	Significant improvement in understanding surgical instruments and techniques	3D simulation combined with printed models enhanced surgical skills and knowledge acquisition	Small sample size, single institution
Shetty et al.	2023	Impact of fully guided implant planning software training on the knowledge acquisition and satisfaction of dental undergraduate students	Controlled experimental study	90 final-year dental students	Virtual implant planning software (VIPS) with lectures, video, and hands-on sessions	Students in the hands-on group (Group C) performed significantly better in knowledge and procedural components	Hands-on training significantly improved performance compared to video and lecture groups	Short-term study, limited to one university, no long-term retention assessment
Elhadidi et al.	2024	The effect of online-teaching and simulated-training during COVID-19 Lockdown on students	Audit and survey	40 dental students	Online and hybrid learning with simulated training	Significant dissatisfaction with online-only training compared to hybrid; higher satisfaction with in-class learning	Students preferred real patients for clinical practice, but hybrid models were acceptable during lockdown	Small sample size, limited to one institution, pandemic-specific context
Hu et al.	2023	Patient-specific 3D printed models for enhanced learning of immediate implant procedures and provisionalization	Controlled study	30 dental students	3D-printed models for immediate implant placement and provisionalization	Significant improvements in understanding of surgical and prosthetically driven procedures	3D models were cost-effective and improved practical and theoretical knowledge	Limited to one case scenario, no soft tissue simulation
Blond et al.	2024	Blended learning compared to traditional learning for the acquisition of competencies in oral surgery by dental students	Randomized controlled trial (RCT)	73 4th-year dental students	Blended learning (combining online resources and face-to-face) vs. traditional learning for oral surgery	Blended learning led to higher satisfaction and improved perceived skills in anesthesia and surgical tooth extraction	Students in the blended group felt more confident in performing complex procedures (e.g., surgical extraction with bone removal), though no significant difference in clinical performance was observed between groups after 6 months	Limited by unequal teaching hours between groups, single institution, and potential bias from peer interaction
Buchbender et al.	2021	Kobra Surgery Simulator—A Possibility to Improve Digital Teaching? A Case-Control Study	Case–control study	49 dental students (third and fourth year) and 10 dentists	Kobra Surgery Simulator for simulating apicoectomy and wisdom tooth extraction	Improvement in practical skills, comparison with conventional plastic models, and introduction of simulation-based teaching	Conventional plastic models were preferred slightly over the simulator; students performed less precisely than dentists	Small sample size; subjective assessment of learning experience; no significant performance differences between students and dentists
Fischer et al.	2023	Introducing a novel educational training programme in dental implantology for pregraduate dental students	Survey-based educational study	94 dental students (3rd–5th years)	Implantology training program consisting of 4 modules (theoretical, practical, and clinical exercises)	High student satisfaction, increased knowledge of implantology, desire for more practical experience	88% of students wanted more hands-on training, with many expressing interest in performing supervised implant procedures	Limited student cohort, results based on student feedback only, no patient outcomes considered
Bock et al.	2020	Flipped OR: A modified didactical concept for a surgical clerkship in Oral and Maxillofacial Surgery	Prospective cohort study	21 dental students	Flipped classroom approach with an e-learning module and clinical clerkship	Significant improvement in test scores after the clerkship; high satisfaction with e-learning and surgical videos	Students found the surgical videos very helpful for understanding procedures; blended learning enhanced knowledge retention	Small sample size; time intervals between tests varied; no control group for comparison with traditional methods
